# Tumor Cell Stemness and Stromal Cell Features Contribute to Oral Cancer Outcome Disparity in Black Americans

**DOI:** 10.3390/cancers16152730

**Published:** 2024-07-31

**Authors:** Saied Mirshahidi, Isabella J. Yuan, Zhong Chen, Alfred Simental, Steve C. Lee, Pedro A. Andrade Filho, Thomas Murry, Feng Zeng, Penelope Duerksen-Hughes, Charles Wang, Xiangpeng Yuan

**Affiliations:** 1 Department of Basic Sciences, School of Medicine, Loma Linda University, Loma Linda, CA 92354, USA; 2 Cancer Center Biospecimen Laboratory, Loma Linda University Medical Center, Loma Linda, CA 92354, USA; 3 Department of Otolaryngology-Head and Neck Surgery, Loma Linda University Medical Center, Loma Linda, CA 92354, USA; 4 Center for Genomics, School of Medicine, Loma Linda University, Loma Linda, CA 92350, USA

**Keywords:** racial disparities, oral cancer, tumor stemness, tumor stroma, post-surgery outcome

## Abstract

**Simple Summary:**

Head and neck cancer disparities in outcomes for Black Americans have been well recognized. However, the specific drivers of the inferior outcomes remain poorly understood. We investigated the biologic features of patient oral cancers and performed a follow-up study of the patient post-surgery recurrences and metastases aiming to explore potential mechanisms that might underpin the poorer outcomes among Black American patients. It was found that high levels of tumor stemness and tumor-promoting stromal characteristics were linked to patient recurrence and metastasis. There were more cases of Black American than White American exhibiting high stemness traits and strong tumor-promoting stromal features associated with tumor recurrences and metastases, although the investigated cases displayed comparable clinical diagnoses. Our findings revealed that the differences in tumor stemness and stromal property among cancers with similar diagnoses contribute to patient outcome disparities.

**Abstract:**

Black Americans (BAs) with head and neck cancer (HNC) have worse survival outcomes compared to the White patients. While HNC disparities in patient outcomes for BAs have been well recognized, the specific drivers of the inferior outcomes remain poorly understood. Here, we investigated the biologic features of patient tumor specimens obtained during the surgical treatment of oral cancers and performed a follow-up study of the patients’ post-surgery recurrences and metastases with the aim to explore whether tumor biologic features could be associated with the poorer outcomes among BA patients compared with White American (WA) patients. We examined the tumor stemness traits and stromal properties as well as the post-surgery recurrence and metastasis of oral cancers among BA and WA patients. It was found that high levels of tumor self-renewal, invasion, tumorigenesis, metastasis, and tumor-promoting stromal characteristics were linked to post-surgery recurrence and metastasis. There were more BA than WA patients demonstrating high stemness traits and strong tumor-promoting stromal features in association with post-surgery tumor recurrences and metastases, although the investigated cases displayed clinically comparable TNM stages and histological grades. These findings demonstrated that the differences in tumor stemness and stromal property among cancers with comparable clinical diagnoses contribute to the outcome disparity in HNCs. More research is needed to understand the genetic and molecular basis of the biologic characteristics underlying the inferior outcomes among BA patients, so that targeting strategies can be developed to reduce HNC disparity.

## 1. Introduction

Head and neck cancer (HNC) is the sixth most common malignancy worldwide [1,2]. It accounted for nearly 900,000 new cases and 458,107 deaths in 2022 [3]. Metastasis and recurrence result in a 5-year survival rate of approximately 50% after surgery, which has not obviously improved over the past decade [4,5]. According to recent cancer statistics, oral cavity and pharynx cancers—the major components of HNC—will account for 54,540 new cases in 2023 and 11,580 estimated deaths in the United States [6]. Epidemiology data have identified disparities between racial groups in HNC [7,8,9,10,11,12]. Outcome estimates reported by the American Cancer Society reflect up to an 18% survival difference between Black American (BA) and White American (WA) patients with oral cavity and pharynx tumors [13]. These findings demonstrated that the disparity in HNC is a significant health issue currently affecting BAs. Recent studies have started to reveal that biologic and genetic factors, besides socioeconomic status and life styles as well as environmental exposure to carcinogens, are most likely contributing to the inferior outcomes in BAs with HNCs [14,15,16,17]. Although personal, environmental, and socioeconomic factors may be drivers of the existing racial disparities in HNC, the biological alterations at play alongside these factors warrant deeper investigation to establish tumor microenvironment (TME) malignant and non-malignant cell features associated with clinical inferior outcomes, and to develop more appropriate treatment strategies to reduce health disparity.

To date, comparative analyses of tumor biological characteristics involved in different racial groups that display diverse clinical outcomes are largely lacking for HNC. The potential roles of tumor cell stemness—the driving force of tumorigenesis and tumor progression [18,19,20,21,22,23,24,25,26,27,28,29]—and tumor stromal features that define either tumor-promoting or tumor-suppressive capabilities associated with tumor recurrence/metastasis in HNC racial disparity are unknown. In this study, the potential impacts of tumor biologic characteristics on patient post-surgery outcomes and on racial disparities in oral cancer were investigated. We tested the hypothesis that tumor cell stemness traits and tumor stromal properties contribute to post-surgery outcome disparities between BA and WA HNC patients because genetic differences between these two groups of patients have been reported [2,30,31,32,33,34], and such differences could impact tumor stemness and stromal features in HNCs. This work, focusing on oral cavity cancer, aimed to investigate the clinical importance of assessing cancer cell stemness and stromal property in indicating patient post-surgical treatment outcomes and to examine the influence of tumor biologic factors on racial disparity in HNC.

## 2. Materials and Methods

### 2.1. Preparation of Primary Cells from Oral Cavity Cancer Tissue

Human oral cavity cancer tissues were obtained intraoperatively after informed consent as approved by the Institutional Review Board at Loma Linda University. We investigated 26 BA and 40 WA tumor specimens in this project. Tumor specimens were washed in PBS, minced with sterile blades, and incubated with collagenase (STEMCELL Technologies, Cambridge, MA, USA) for 1–2 h at 37 °C. After enzymatic digestion, the samples were filtered through 40 μm cell strainers and mixed with ammonium chloride solution for 5 min to lyse red blood cells. The prepared oral cancer cells were resuspended in serum-free cancer stem cell (CSC) culture medium as described in a previous study [35]. In the serum-free conditions, cells were growing in low-attachment 12-well or 24-well plates as spheres. For serial passaging, spheres were collected by gentle centrifugation and dissociated enzymatically with 0.05% trypsin/EDTA. The dissociated cells were passed through a 40 μm mesh filter to obtain single cell suspensions. Single cells were plated at 5000 cells/well (for 12-well plate) in the low-attachment plates to generate the next passage spheres. All the investigated primary cells were derived from the patient tumor specimens. The detailed information of these specimens relevant to the current study presentation are shown in Table 1.

### 2.2. Limiting Dilution Analysis (LDA)

For the in vitro (limiting dilution) sphere-formation assay, tumor spheres were dissociated into single cells, and viable cells were seeded into 96-well plates at different cell doses. For each cell dose, at least 12 wells were seeded with cells, and for the lower cell doses, generally 96 wells were plated for an individual cell dose. Three to five weeks later, wells containing spheres were scored, and the number of positive wells was used to calculate the frequency of sphere-forming units using the ELDA software provided by the Walter and Eliza Hall Institute [36]. For the transwell culture LDA, tumor stromal cells were seeded into the top insert (1.0 μm pore size) chambers while tumor cells were plated in the bottom chambers.

### 2.3. Tumor Cell Invasion Assay

Tumor cell invasion was assessed via a Boyden chamber assay with 24-well plate inserts (8.0 μm pore size) pre-coated with standard Matrigel (Corning, Tewksbury, MA, USA). The subsequent detection of invasive cells was performed with either crystal violet or hematoxylin-eosin staining. The bottom chambers were seeded with 0.5–1 × 10^5^ of tumor or non-tumor tissue-derived stromal cells in serum-containing medium and allowed to grow for 12–24 h before setting up the top insert chambers. The top chambers were seeded with 2.5 × 10^4^ tumor tissue-derived or tumor sphere-derived single cells resuspended in serum-free CSC culture medium. The seeded cells were allowed to invade for 22 h. Upon the completion of the 22 h invasion, non-invasive cells in the top chamber were removed with cotton swabs, and invasive cells on the insert bottom membrane were fixed in 1% paraformaldehyde and processed for staining. Stained cells were scored under the microscope. Independent invasion experiments were performed in duplicate or triplicate wells.

### 2.4. Xenograft Tumor Establishment

Tumor tissue- or tumor sphere-derived single cells were serially diluted and injected submucosally into the tongue of NOD/SCID mice (aged 6–8 weeks) as orthotopic xenografts. The injected animals were monitored for tumor formation. When xenograft tumor volumes reached approximately 0.5 cm in diameter, tumors were harvested and prepared for primary cell cultures as described above in the patient specimen preparation. Tumor spheres isolated from xenografts were used for CSC in vitro sphere formation and in vivo tumorigenesis assays. For the in vivo LDA, tumor cells either from tumor tissues or from the cultured tumor spheres were processed into single cells and diluted serially to desired cell doses. Viable cells were injected submucosally into NOD/SCID mice. Tumor formation was monitored once a week. The number of tumors formed out of the number of sites injected was scored to determine the frequency of tumor-initiating cells (TICs) calculated using the ELDA software [36]. Lymph node metastasis was evaluated at the end point of the experiments. For the studies of assessing tumor stromal cell influence on tumorigenesis, tumor cells and stromal cells were co-injected submucosally. Viable cells were determined using trypan blue exclusion analysis. The percentage of trypan blue negative cells was used to calculate the frequencies of sphere-forming cells and TICs. All mouse study protocols were approved by the Institutional Animal Care and Use Committee at Loma Linda University.

### 2.5. Co-Culturing of Tumor Stromal Cells and T Cells for Immune Regulation Assay

Tumor stromal cells were isolated from the tumor tissue-derived single cells through the depletion of EpCAM^+^ cells as the negative selection strategy (CD326 (EpCAM) MicroBeads, human, Miltenyi Biotec, San Diego, CA, USA). The isolated stromal cells were seeded into 96-well plates (10^4^ cells/well) and cultured around 6 h for cell adherence in a 37 °C incubator. CD3^+^ T cells from peripheral blood mononuclear cells (PBMCs) of healthy donors were isolated using indirect magnetic labeling cell separation (REAlease CD3 MicroBead Kit, human, Miltenyi Biotec). CD4^+^ and CD8^+^ T cells were isolated from the CD3^+^ T cells by positive selection using a human CD4^+^ or CD8^+^ T cell isolation kit (Miltenyi Biotec). The isolated CD4^+^ and CD8^+^ T cells were initially incubated with carboxyfluorescein succinimidyl ester (CFSE) for 15 min at 37 °C, and then added to tumor stromal cell-seeded 96-well plates (10^5^ cells/well). The co-culture system was subsequently incubated with anti-CD3 and anti-CD28 antibodies (250 ng/mL) and IL-2 (10 ng/mL). Cells were harvested for flow cytometric analyses after 3 days of coculturing. For cell surface marker staining, antibodies were used (anti-CD4, anti-CD8, and anti-PD-1) with the staining process in the dark. For intracellular staining, cells were stimulated with eBioscience™ Cell Stimulation Cocktail (Thermo Fisher Scientific, Waltham, MA 00-4975-93) according to the manufacturer’s instructions, fixed and permeabilized, and then stained with intracellular molecule-targeting antibodies against GzmB and Foxp3. After washing, cells were analyzed with MACSQuant Analyzer 10 (Miltenyi Biotec, San Diego, CA, USA) and FlowJo software (Treestar, Ashland, OR, USA).

### 2.6. Statistics

Limiting-dilution analyses for frequency determinations of sphere-forming cells and cancer-initiating cells, as well as the corresponding *p* values, were performed using ELDA software, which took into account whether the assumptions for LDA were met [36]. The data and error bars report the mean ± SD. For comparisons of immune molecule expression and tumor cell invasion, the two-tailed Student’s *t*-test was performed between two groups and a difference was considered statistically significant with *p* < 0.05.

## 3. Results

### 3.1. Oral Cancers in Comparable TNM Stages and Histologic Grades Constitute Distinct Features of Cancer Stemness

**Figure 1:**
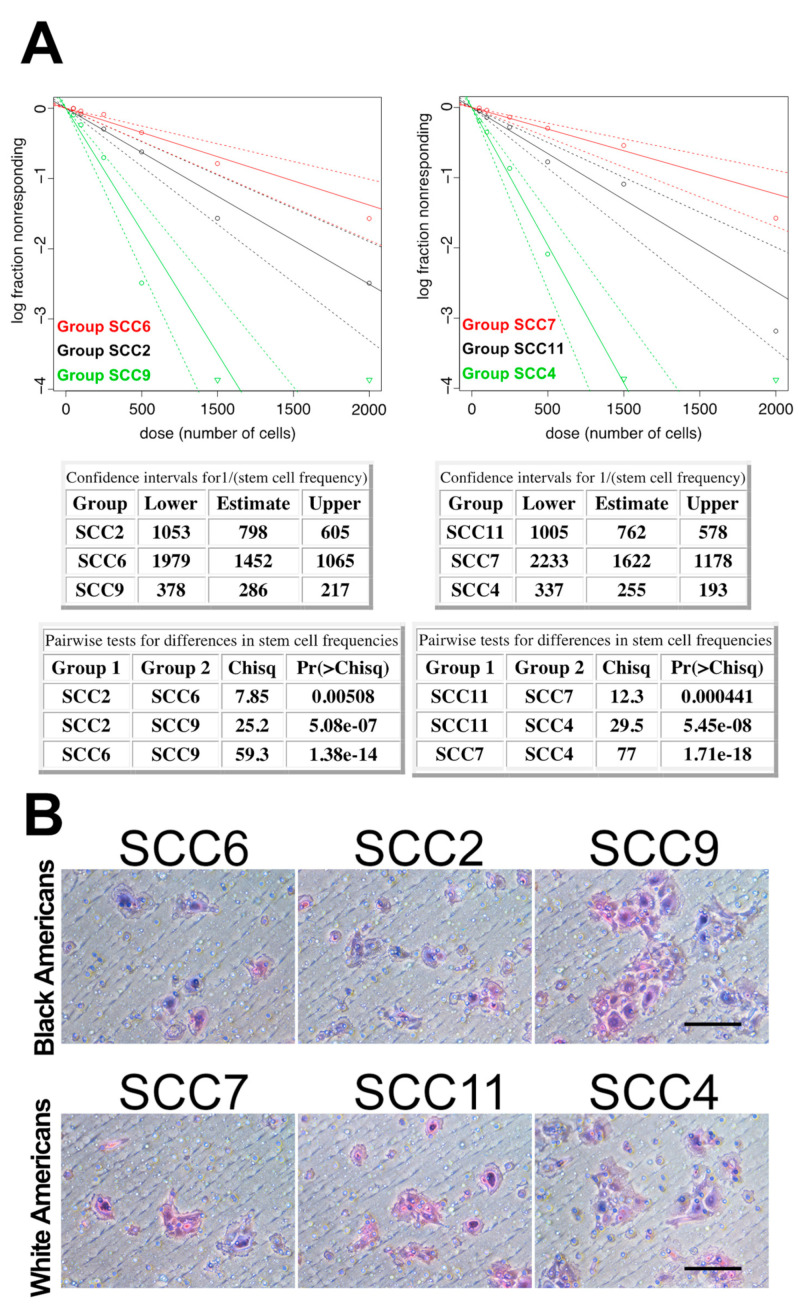
Oral cancers with comparable TNM stages and histologic grades demonstrated different features of cancer cell stemness. (**A**) The tumor cells of individual oral cancers showed different capacities of tumor sphere formation within the patient specimen-derived primary cell cultures. Representative cases of both Black American (**left**) and White American (**right**) patient specimens are shown. (**B**) The tumor cells of individual oral cancers demonstrated different invasive activities. Representative cases of both Black American (**top panel**) and White American (**bottom panel**) patient specimens are shown. Scale bar: 100 μm.

**Figure 2:**
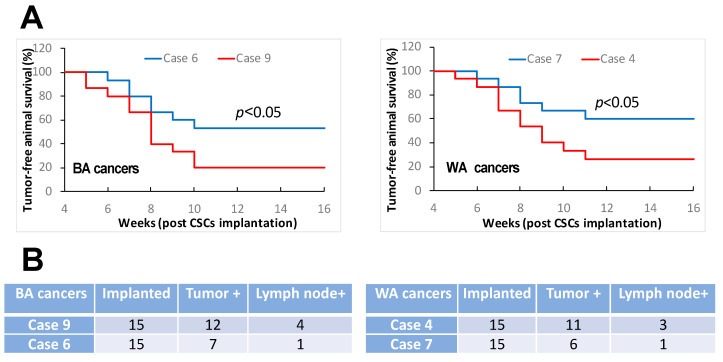
Oral cancers with comparable TNM stages and histologic grades demonstrated different features of tumorigenesis and metastasis. The tumor cells of individual oral cancers had different capacities of tumor initiation (**A**) and lymph node metastasis (**B**) in orthotopic xenograft models. Representative cases from Black American (BA, left graph and left table) and White American (WA, right graph and right table) patient specimens are shown. Case 6: SCC6; Case 9: SCC9; Case 4: SCC4; Case 7: SCC7.

It has been well recognized that BAs with HNCs have poorer survival compared to WA HNC patients [7,8,9,10,11,12,13]. Although the socioeconomic status, access to care, life styles, and genetic features have been suggested as potential factors contributing to the inferior outcomes of HNCs in BA patients [14,15,16,17], there is currently a lack of evidence on whether the BA and WA HNCs have distinct biologic characteristics that significantly contribute to the survival differences between the two groups of patients. We investigated clinical oral cancer specimen’s stemness traits aiming to explore biological features that might be relevant to potential mechanisms underpinning the different survival rates among BA and WA patients. We selectively recruited the tumor cases so that all the tumors under investigation had comparable tumor–node–metastasis (TNM) stages and histologic grades upon their surgical treatment in which the tumor specimens were harvested for our investigations. In addition, only human papillomavirus negative (HPV^−^) tumors were included in this study. With such criteria for subject recruitment, we aimed to avoid certain potential factors that could confound our findings. We focused on selected study subjects in this investigation because HNCs were generally diagnosed in more advanced stages among BA compared with WA patients and HPV-positive rates are different between individual racial groups of HNC patients. Both tumor stage and HPV status can impact cancer stemness. It was found that a proportion of BA oral cancer cases showed significantly higher tumor cell sphere formation capacities than the other BA cases as well as the majority of WA cases (Figure 1A). It was also discovered that a minority of WA oral cancer cases demonstrated higher capacities of tumor cell sphere formation compared to the majority of the cases (Figure 1A). The sphere formation efficiencies observed in the special proportion of BA cases and in the identified minority of WA cases were comparable (Figure 1A). As tumor cell sphere-forming capacity represents CSC’s self-renewal abilities that essentially contribute to tumorigenesis and tumor progression [18,19,20,21,22,23,24,25,26,27,28,29,37,38], the different capacities of sphere formation discovered among individual oral cancer cases consisting of similar TNM stages and histologic grades represented a significant result warranting further investigations. Tumor cell invasive activity is associated with tumor progression and metastasis [39,40,41,42,43,44,45]. We therefore assessed the invasive potentials of tumor cells dissociated from individual cancer specimens. It was found that the tumors with high sphere formation capacities showed superior invasive activities compared to the tumors with low sphere formation capacities for both BA and WA patient cases (Figure 1B). These findings revealed differences in tumor invasive potentials between the individual cancers that were characterized as comparable in TNM stages and histologic grades. Furthermore, we investigated the tumorigenic and metastatic capacities of the above tumor specimens. For these investigations, we performed an orthotopic implantation of the patient specimen-derived primary cells using NOD/SCID mice. It was observed that the tumor specimens with high levels of tumor cell sphere formation and invasion demonstrated stronger tumorigenic activities compared to the specimens with low levels of tumor cell sphere formation and invasion (Figure 2A). In addition, the stronger tumorigenic activities were associated with increased likelihood of lymph node metastasis development, as around 10–30% vs. 0–10% of the orthotopically implanted mice developed lymph node metastases in the tumor specimens with stronger vs. the specimens with weaker tumorigenic activities (Figure 2B). Collectively, the above data showed that oral cancers could constitute distinct cancer stemness traits although clinically displaying comparable TNM stages and histologic grades, and that the variable capacities of cancer cell self-renewal, invasion, and tumorigenesis/metastasis among the clinically comparable individual oral cancers were the biological characteristics featured in BA and WA patients.

### 3.2. TME Stromal Cells of Individual Oral Cancers Have Different Capabilities of Affecting CSC Stemness

**Figure 3:**
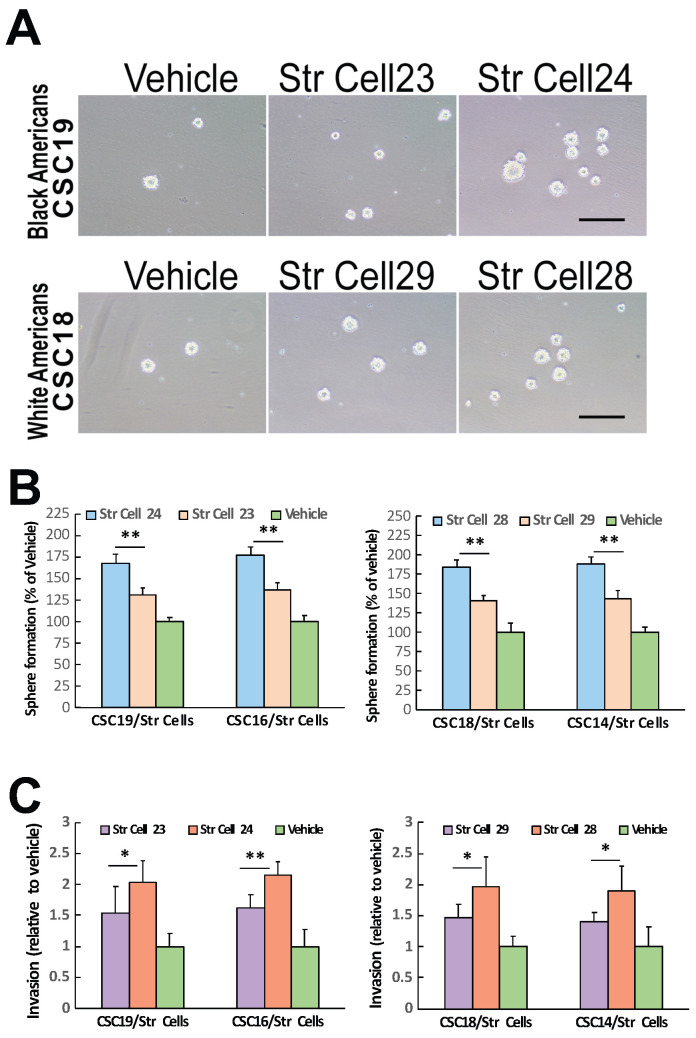
Tumor stromal cells from individual oral cancers with comparable TNM stages and histologic grades demonstrated different capacities of affecting cancer stem cell (CSC) functions. (**A**) Tumor stromal cells isolated from individual cases of Black American (**top**) and White American (**bottom**) oral cancers demonstrated the effects of influencing CSC sphere formation in transwell co-cultures. Representative cases are shown. Scale bar: 50 μm; Str Cell: tumor stromal cells isolated from individual SCC specimens; Vehicle: culture medium alone. (**B**) Stromal cells from different tumors showed distinct capacities of affecting individual tumor-derived CSC sphere formation potentials compared with the vehicle in both Black American (**left**) and White American (**right**) oral cancer cases. (**C**) Tumor stromal cells from different cases of Black American (**left**) and White American (**right**) oral cancers showed different capacities of enhancing individual tumor-derived CSC invasive activities compared with the vehicle. * *p* < 0.05; ** *p* < 0.01.

**Figure 4:**
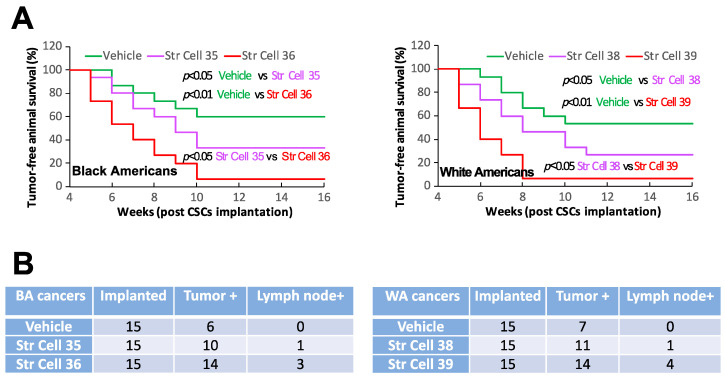
Tumor stromal cells from individual oral cancers with comparable TNM stages and histologic grades demonstrated different capacities of affecting cancer stem cell (CSC) tumorigenesis and metastasis. Tumor stromal cells from different cases of oral cancers demonstrated distinct capacities of enhancing CSC tumor initiation (**A**) and lymph node metastasis (**B**) in orthotopic xenograft models. Representative cases from Black American (**left**) and White American (**right**) patient specimens are shown. Str Cell: tumor stromal cells isolated from individual SCC specimens; Vehicle: saline; BA: Black American; WA: White American.

Increasing studies have demonstrated that tumor stromal cells can affect malignant cells to exert either tumor-promoting or tumor-suppressing effects [46,47,48,49,50,51,52]. To investigate whether TME stromal cells of individual oral cancers that exhibited comparable TNM stages and histologic grades could exert comparable effects on CSC biological activities, we initially tested the influence of TME stromal cells on CSC self-renewal potentials. Co-culturing TME stromal cells with the CSCs isolated from different tumor specimens enhanced CSC sphere-formation efficiencies (Figure 3A,B). The sphere-enhancing effects of TME stromal cells were highly variable among the individual samples investigated. The stromal cells from tumors with high levels of tumor cell stemness traits (self-renewal, invasion, and tumorigenesis/metastasis) demonstrated higher potentials of enhancing the co-cultured CSCs’ sphere formation compared to the stromal cells from the tumors with low levels of tumor cell stemness traits (Figure 3A,B). In addition, stromal cells from the tumors with high levels of tumor cell stemness traits demonstrated higher capacities of promoting the invasive activities of CSCs in comparison with the stromal cells from the tumors with low levels of tumor cell stemness traits (Figure 3C). Furthermore, TME stromal cells from tumors with high levels of cancer cell stemness traits showed higher capacities of promoting the tumor initiation and metastasis of the CSCs derived from different tumor specimens compared to the stromal cells from tumors with low levels of cancer cell stemness traits (Figure 4). All together, these results revealed that stromal cells of the different individual tumors with comparable TNM stages and histologic grades could have distinct capacities of affecting cancer cell biologic activities and influencing tumorigenesis/metastasis.

### 3.3. TME Stromal Cells of Individual Oral Cancers Have Variable Potentials of Suppressing T Cell Functionality

**Figure 5:**
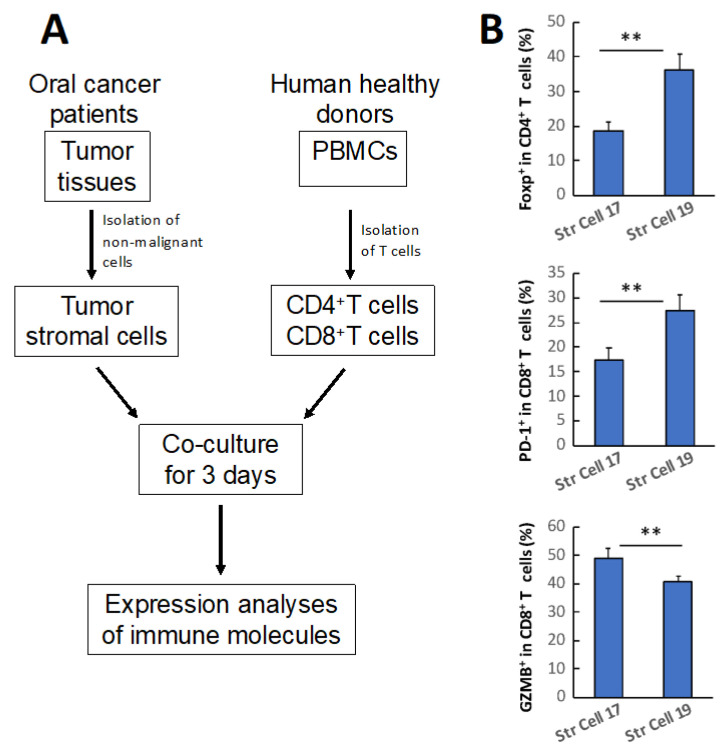
Tumor stromal cells from individual oral cancers with comparable TNM stages and histologic grades demonstrated diverse capacities of regulating immune cell functionality. (**A**) Schematic diagram depicting the strategy of assessing tumor stromal cells interacting with immune cells in regulating immune cell function. (**B**) Tumor stromal cells isolated from different cases of oral cancers showed their capacities of altering the proportions of Foxp3^+^ cells within total CD4^+^ T cells (top), and the proportions of PD-1^+^ (middle) and GzmB^+^ (bottom) cells, respectively, in total CD8^+^ T cells. PBMCs: peripheral blood mononuclear cells; Str Cell: tumor stromal cells isolated from individual SCC specimens; ** *p* < 0.01.

Tumor stromal cells have been recognized as being capable of regulating immune cell function to influence tumor development and progression [53,54,55]. We investigated the influences of oral cancer stromal cells on T cell functionality. The purpose was to explore whether stromal cells of the individual oral cancers in comparable TNM stages and histologic grades have similar capacities of regulating immune cell activities. We isolated TME stromal cells from different individual cancer specimens and co-cultured the cells with T cells derived from PBMCs of healthy human donors. We assessed the immune response molecules in the CD4^+^ and CD8^+^ T cells. It was found that stromal cells from the tumors exhibiting a high capacity of tumor cell self-renewal, invasion, and tumorigenesis/metastasis had stronger potentials of suppressing T cell function in comparison with the stromal cells from the tumors exhibiting low capacities of tumor cell self-renewal, invasion, and tumorigenesis/metastasis (Figure 5). These immune-suppressing effects were evidenced as increased proportions of Foxp3^+^ cells and PD-1^+^ cells in the total CD4^+^ and CD8^+^ T cells, respectively, and as decreased proportions of GzmB^+^ cell in total CD8^+^ T cells (Figure 5). These results demonstrated that TME stromal cells of individual oral cancers could have different capacities of impacting immune cell functionality, although the individual cancers might display comparable TNM stages and histologic grades clinically, and that tumor stromal cell immune-suppressive capacities were linked to the tumor’s cancer cell stemness traits.

### 3.4. Tumor Cell Stemness and Tumor Stromal Cell Property Are Linked to Oral Cancer Post-Surgery Recurrence and Metastasis

**Figure 6:**
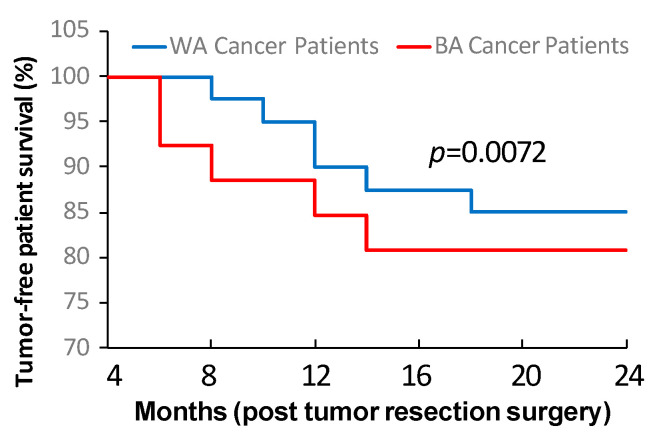
Patient relapse-free survival after tumor resection surgery. The oral cancers with comparable TNM stages and histological grades demonstrated different tumor-free survivals after tumor resection as the BA patients exhibited lower relapse-free survival compared to the WA patients. BA: Black American; WA: White American; TNM: Tumor–node–metastasis.

The results described above demonstrated that tumor stemness traits and stromal features could vary significantly among individual oral cancers that displayed comparable TNM stages and histologic grades. Based on these data, we next investigated potential links between the findings achieved in the tumor cell stemness as well as stromal cell property analyses and the patient tumor recurrence and/or metastasis status in post-surgery follow-up assessments. We aimed to explore whether the distinct biologic features contributed to the differences in clinical outcomes among individual oral cancer cases that exhibited comparable histopathological characteristics. With the post-surgery follow-up study, it was found that the tumor cases consisting of high levels of sphere formation efficiencies (<1:500) and TIC frequencies (<1:10,000) inevitably developed recurrences and/or lymph node metastases after initial surgical treatments (Table 2). In addition, the tumor cases consisting of high sphere formation efficiencies as well as high TIC frequencies plus the development of post-surgery recurrences and/or metastases had elevated capacities of stromal cells in enhancing CSC stemness traits and suppressing T cell functionality. In contrast, the tumor cases consisting of low levels of sphere formation efficiencies (>1:1000) and TIC frequencies (>1:100,000) did not have signs of post-surgery tumor recurrence and/or metastasis during the clinical follow-up studies (Table 2). Also, these tumor cases had low capacities of stromal cells in promoting CSC stemness traits and suppressing T cell functionality. These findings revealed the association of tumor stemness traits and stromal features with patients’ potentials of developing post-surgery recurrence and/or metastasis, demonstrating that tumor cell stemness and stromal cell property represent the crucial factors influencing patient clinical outcomes. More importantly, we observed that more BA than WA oral cancer patients showed high levels of tumor stemness traits, tumor-promoting stromal features, and post-surgery recurrence/metastasis formation (Table 2). Such differences between the BA and WA patient groups proved that tumor cell stemness and tumor stromal properties can significantly impact clinical outcomes of oral cancer. Further analysis of tumor-free survival revealed that BA patients had significantly lower tumor-free survival compared to WA patients after the tumor resection by initial surgery (Figure 6). These investigated tumors had comparable TNM stages and histologic grades in both BA and WA patients. Together, these data demonstrated that tumor stemness and stromal property impacted oral cancer outcomes, and that more BA than WA patients had high levels of tumor stemness traits and tumor-promoting stromal features, which might contribute to HNC disparities.

## 4. Discussion

Disparity in HNC has been well documented. However, factors contributing to the inferior outcomes of HNCs among BAs have not been sufficiently understood, which makes targeting HNC disparity in BAs a significant challenge. Numerous studies suggested that socioeconomic status, access to care, life styles, and genomic background are potential factors contributing to the low survival rates of HNC in BA patients compared with the patients of other racial groups [7,8,9,10,11,12,13]. Interestingly, recent research findings suggested that distinctions in tumor molecular features between the patients of different racial groups could be potentially underpinning the outcome disparities in HNCs [14,15,16,17]. Our present study discovered differences in oral cancer biological characteristics between the individual patients who had comparable features in clinical diagnostic evaluation upon initial surgical treatments. These biological characteristic differences were linked to distinctions in the potentials of tumor recurrence and metastasis. Our data revealed that the differences in tumor biologic features could vary enormously among BA and WA oral cancer patients, which might account for potential mechanisms underlying the lower survival rates observed in BA in comparison with WA HNC patients.

Cancer cell stemness has been identified as the driving force of tumor initiation and progression [18,19,20,21,22,23,24,25,26,27,28,29]. Our investigation of patient tumor specimens revealed distinctions in cancer cell stemness traits among oral cancers that had been characterized as comparable in TNM staging and histologic grading evaluations. A proportion of BA and a minority of WA cases had much higher levels of tumor cell stemness traits compared with other cases among the investigated specimens. These data indicate that heterogeneities of tumor stemness in oral cancer might lead to distinct clinical outcomes in patients who displayed similar features in clinical diagnostic analyses. In addition to the essential roles of tumor cell stemness, tumor stromal cells also influence tumor development and progression [46,47,48,49,50,51,52], which can critically impact clinical outcomes of cancer patients [39,41,44,45]. Our studies found that tumor stromal cells from the tumors with high levels of cancer stemness traits demonstrated elevated capacities of enhancing CSC stemness and suppressing T cell functionality compared to the tumor stromal cells from the tumors with low tumor stemness traits. These findings suggest that tumor stromal cells could have different potentials of regulating oral cancer development and progression depending on the properties of the tumor cells in which the stromal cells were interacting with in a microenvironment. With the post-surgery follow-up investigation, we found that high levels of tumor stemness traits and strong tumor-promoting stromal features were linked to patient post-surgery tumor recurrences/metastases. These data are consistent with previous reports regarding the potential roles of tumor stemness and tumor-promoting stroma in disease progression [56,57,58,59], and suggest the relevance of assessing tumor stemness and stromal property to the development of appropriate therapeutic strategies to improve patient clinical outcomes.

More significantly, our study revealed that the proportions of the cases with high levels of tumor stemness traits and tumor-promoting stromal features were larger among BA patients than among WA patients. Superior stemness traits and stronger tumor-promoting stromal characteristics were associated with high potentials of post-surgery tumor recurrence and metastasis. These findings reveal that more BA than WA patients of oral cancer have been experiencing tumor recurrences and/or metastases, which is similar to what has been suggested in a very recent study investigating HNC therapeutic clinical trials [60]. The higher number of cases subject to the development of post-surgery recurrences and metastases in BA than in WA patients might represent the potential mechanisms that underpin the inferior outcomes of HNC in BAs.

There are limitations in the current study since our investigations covered a relatively limited sample size (i.e., only selected TNM stages, histologic grades, and HPV^−^ status being included). Further validation of the findings across larger cohorts is required to support the generalizability of these findings. In addition, future work to understand the biologic factors at molecular levels that contribute to racial disparities in HNCs are needed.

In sum, our study discovered distinctions in tumor cell stemness and tumor stromal property between individual tumors. Such distinctions were linked to tumor recurrence and metastasis. The presence of more BA than WA patients with oral cancer characterized by high tumor stemness and strong tumor-promoting stroma in association with post-surgery tumor recurrence and metastasis might account for, at least partially, the mechanisms underlying the inferior outcomes of HNC among BAs. Our findings pave the way for potentially developing appropriate strategies targeting HNC disparities that are currently affecting BAs.

## 5. Conclusions 

There are more BA than WA oral cancer patients with high-level cancer stemness and tumor-promoting stroma. Cancer cell stemness and stromal cell property are crucial factors contributing to oral cancer post-surgery outcome disparity.

## Figures and Tables

**Table 1:** Detailed information about patient specimens investigated.

Sample ID	Primary Site	TNM Stage	Tumor Grade	Race	Sex	Age at Surgery	Smoking History	HPV Status	Recurrence	Lymph Node
SCC2	lip	T2N0M0	G2	BA	Male	51	Yes	Negative	No	No
SCC6	gingival	T2N1M0	G2	BA	Male	63	No	Negative	No	No
SCC9	tongue	T2N1M0	G2	BA	Female	68	Yes	Negative	Yes	Yes
SCC16	floor of mouth	T3N0M0	G3	BA	Male	66	Yes	Negative	No	No
SCC17	tongue	T2N0M0	G2	BA	Male	49	No	Negative	No	No
SCC19	floor of mouth	T2N0M0	G3	BA	Male	55	No	Negative	Yes	No
SCC23	buccal mucosa	T3N0M0	G2	BA	Female	63	Yes	Negative	No	No
SCC24	gingival	T2N1M0	G2	BA	Male	72	Yes	Negative	Yes	Yes
SCC35	gingival	T3N0M0	G3	BA	Male	59	No	Negative	No	No
SCC36	tongue	T3N0M0	G3	BA	Male	68	Yes	Negative	Yes	Yes
SCC4	floor of mouth	T2N1M0	G3	WA	Male	53	Yes	Negative	Yes	Yes
SCC7	hard palate	T2N0M0	G2	WA	Female	74	No	Negative	No	No
SCC11	gingival	T3N0M0	G3	WA	Male	70	Yes	Negative	No	No
SCC14	buccal mucosa	T2N1M0	G2	WA	Male	65	Yes	Negative	No	No
SCC18	tongue	T2N0M0	G3	WA	Male	46	No	Negative	No	No
SCC28	tongue	T2N1M0	G3	WA	Female	74	Yes	Negative	Yes	Yes
SCC29	lip	T2N0M0	G2	WA	Male	81	No	Negative	No	No
SCC38	floor of mouth	T3N0M0	G3	WA	Female	69	Yes	Negative	No	No
SCC39	gingival	T3N0M0	G2	WA	Male	77	Yes	Negative	Yes	No

**Table 2:** Oral cancers showed different cancer stemness and recurrence/metastasis. Individual oral cancers with comparable TNM stages and histologic grades showed different capacities of cancer stemness and post-surgery recurrences/metastases. SFE: Sphere-formation efficiency; TICF: Tumor-initiating cell frequency; TNM: Tumor–node–metastasis; +: Recurrence/metastasis-positive; −: Recurrence/metastasis-negative.

		Patients Cases	Cases with SFE 1:100–1:5000			Cases with TICF 1:5000–1:500,000			TNM Stage		Grade_		Sex		Smoking History	
			>1:1000	1:500–1:1000	<1:500	>1:100,000	1:10,000 1:100,000	<1:10,000	T2	T3	G2	G3	Male	Female	Yes	No
**Black American cancer**	Recurrence/ metastasis −	21	14	7	0	12	9	0	14 66.7%	7 33.3%	13 61.9%	8 38.1%	18	3	19	2
	Recurrence/ metastasis +	5	0	1	4	0	2	3	3 60%	2 40%	3 60%	2 40%	4	1	5	0
**White American cancer**	Recurrence/ metastasis −	34	29	5	0	27	7	0	23 67.6%	11 32.4%	22 64.7%	12 35.3%	28	6	30	4
	Recurrence/ metastasis +	6	0	2	4	0	3	3	3 50%	3 50%	4 66.6%	2 33.4%	5	1	5	1

## Data Availability

The original contributions presented in the study are included in the article material, further inquiries can be directed to the corresponding author.
